# Supplementary figures and images for: New Isolation of Ponticelli III Virus (Bunyavirales: Phenuiviridae) in Emilia-Romagna Region, Italy
Source: Viruses. 2023 Feb 2;15(2):422. doi: 10.3390/v15020422 (PMC9964127; doi:10.3390/v15020422)

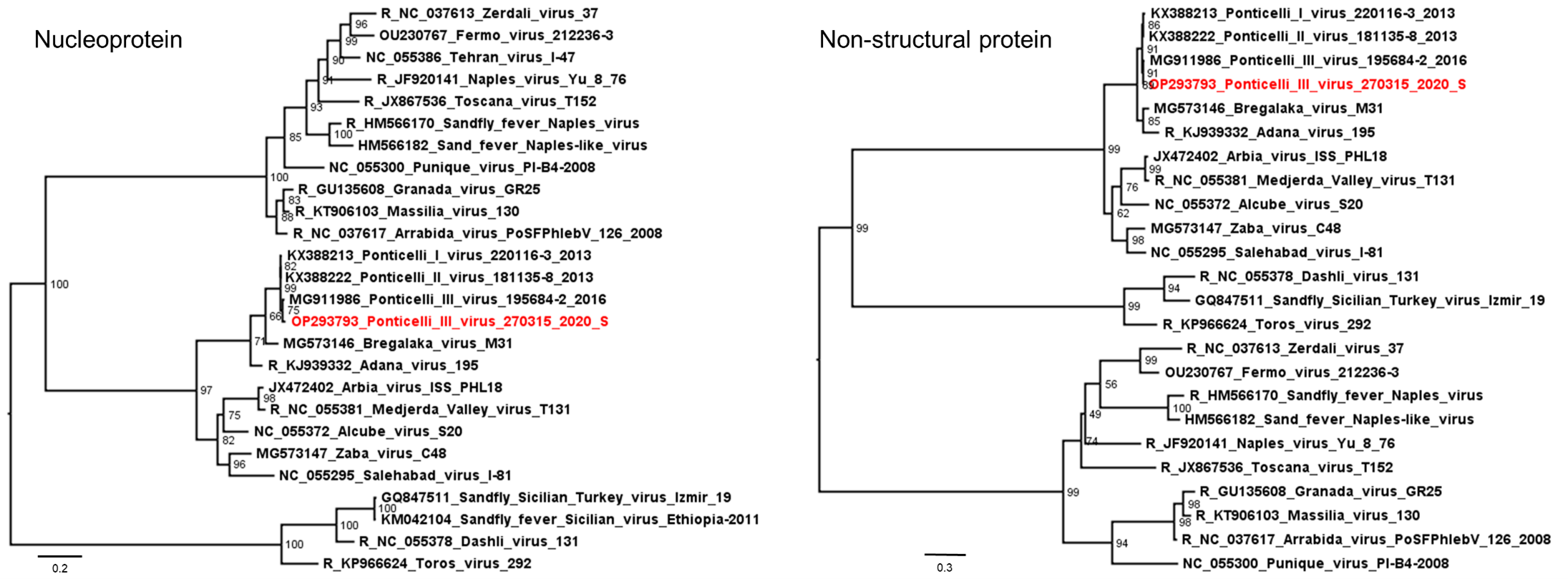

Supplement: Supplementary file 1 [file viruses-15-00422-s001.zip › viruses-1998451-supplementary.tif]
